# Interaction among *Saccharomyces cerevisiae* pheromone receptors during endocytosis

**DOI:** 10.1242/bio.20146866

**Published:** 2014-03-27

**Authors:** Chien-I Chang, Kimberly A. Schandel, Duane D. Jenness

**Affiliations:** Department of Microbiology and Physiological Systems, University of Massachusetts Medical School, 55 Lake Avenue North, Worcester, MA 01655, USA; * Present address: Department of Cancer Biology, University of Massachusetts Medical School, 364 Plantation Street, Worcester, MA 01655, USA.; ‡ Present address: Department of Natural Sciences, Assumption College, 500 Salisbury Street, Worcester, MA 01609, USA.

**Keywords:** G-protein coupled receptors, Receptor oligomers, Ligand-mediated endocytosis

## Abstract

This study investigates endocytosis of *Saccharomyces cerevisiae* α-factor receptor and the role that receptor oligomerization plays in this process. α-factor receptor contains signal sequences in the cytoplasmic C-terminal domain that are essential for ligand-mediated endocytosis. In an endocytosis complementation assay, we found that oligomeric complexes of the receptor undergo ligand-mediated endocytosis when the α-factor binding site and the endocytosis signal sequences are located in different receptors. Both *in vitro* and *in vivo* assays suggested that ligand-induced conformational changes in one Ste2 subunit do not affect neighboring subunits. Therefore, recognition of the endocytosis signal sequence and recognition of the ligand-induced conformational change are likely to be two independent events.

## INTRODUCTION

G protein-coupled receptors (GPCRs) are cell-surface receptors that are present in all eukaryotic organisms. They mediate cellular responses to a wide variety of extracellular ligands and conditions (e.g. hormones, neurotransmitters, odorants and light), and their activity is regulated by covalent modification, by interacting proteins and by membrane protein trafficking (Jean-Alphonse and Hanyaloglu, 2011; Magalhaes et al., 2012). Each receptor contains seven transmembrane-spanning domains, and individual receptors are assembled into multimeric complexes (Salon et al., 2011). Upon ligand binding, the receptor undergoes a conformational change that results in receptor endocytosis in addition to the activation of the heterotrimeric G protein and the consequent signal transduction events. The ways in which signal transduction and membrane protein traffic are related to the oligomeric state of the receptors remain poorly understood.

The α-factor receptor encoded by the *STE2* gene of *Saccharomyces cerevisiae* is a GPCR that mediates the responsiveness of haploid cells of the **a** mating type to the α-factor peptide produced by haploid cells of the α mating type. The receptors are endocytosed constitutively, and the endocytosis rate increases roughly ten fold upon α-factor binding (Jenness and Spatrick, 1986; Schandel and Jenness, 1994). Endocytosis is associated with phosphorylation and ubiquitination of a specific endocytosis signal sequence located in the cytoplasmic C-terminal domain of the receptor (Hicke et al., 1998). Both physiological and biochemical evidence indicates that α-factor receptors form homomultimers in the plasma membrane: α-factor induced internalization of receptor sites is more rapid than internalization of α-factor itself (Jenness and Spatrick, 1986), endocytosis-defective receptors and endocytosis-proficient receptors are co-internalized (Overton and Blumer, 2000; Yesilaltay and Jenness, 2000), differentially-tagged receptors are co-immunoprecipitated (Yesilaltay and Jenness, 2000), fluorescence resonance energy transfer (FRET) occurs between receptors tagged with different fluorescent proteins (Overton and Blumer, 2000), and mutant receptors containing cysteine residue substitutions form inter-protein disulfide cross-links (Wang and Konopka, 2009; Uddin et al., 2012). Recent FRET measurements using a spectrally resolved two-photon microscope are consistent with α-factor receptor multimers containing at least four protomeric units arranged in a parallelogram-shaped tetramer (Raicu et al., 2009). Contact points between protomers have been inferred using FRET measurements of cells expressing receptor fragments (Overton and Blumer, 2002; Overton et al., 2003) and using cysteine–disulfide cross-linking (Wang and Konopka, 2009; Uddin et al., 2012; Umanah et al., 2011), implicating the first and fourth transmembrane spanning domains and transmembrane domains flanking the third intracellular loop. Multiple contact sites between receptors are consistent with the higher-order multimeric structures indicated by spectrally resolved two-photon microscopy (Raicu et al., 2009). Thus far, no communication between protomers within α-factor receptor multimers has been identified in that α-factor binding is non-cooperative (Jenness et al., 1986) and α-factor-binding-defective receptors are unable to complement signaling-defective receptor mutants *in vivo* (Chinault et al., 2004).

This study investigates cooperation among α-factor receptor protomers during endocytosis. Specifically, we ask whether the binding of α-factor to one protomer can utilize the endocytosis signal sequence located in a different protomer of the multimeric complex to stimulate ligand-induced endocytosis. This question pertains to the ability of α-factor-induced conformational changes to spread among the protomers within the complex and to the possibility that the endocytosis signal sequence requires a ligand-induced change in order to interact with the endocytic apparatus. We found that oligomeric complexes of the receptor undergo ligand-mediated endocytosis when the α-factor binding site and the endocytosis signal sequences are located in different protomers, indicating that individual receptors cooperate to provide different receptor functions in *trans*. However, three different assays showed that binding of α-factor to one protomer does not influence the conformational state of neighboring protomers, suggesting that ligand-induced conformational changes and recognition of the endocytosis signal are independent events associated with different regions of the receptor. As a metaphor, α-factor receptors are train passengers that travel as a group. Even without a ticket (endocytosis signal sequence), one member of the group can recognize the train (bind α-factor), board the train (interact with the endocytic machinery) and help his companions board (using subunit interactions). At some point, the conductor will check that at least one member of the group possesses the group's ticket (endocytosis signal sequence) before the train leaves the station (move from the plasma membrane to the vacuole).

## RESULTS

### Complementation between two mutant Ste2 subunits during endocytosis

**Fig. 1.**
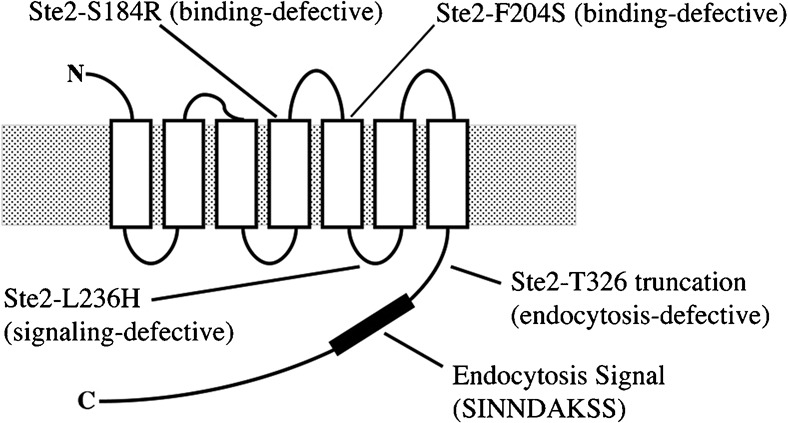
Positions of selected sites in the receptor structure. Schematic representation of the α-factor receptor shows the seven transmembrane domains (boxes) embedded in the cellular membrane (stippled) and joined by the extracellular N-terminal domain, the cytoplasmic C-terminal domain, the three extra-cellular loops and the three cytoplasmic loops. Ste2-S184R and Ste2-F204S receptors fail to bind α-factor (Dosil et al., 2000; Yesilaltay and Jenness, 2000). Ste2-L236H receptors bind α-factor and undergo ligand-mediated endocytosis, but they fail to generate intracellular signals that lead to cell division arrest and transcriptional activation (Schandel and Jenness, 1994). Ste2-T326 receptors are truncated at residue 326 and fail to undergo ligand-mediated endocytosis (Konopka et al., 1988; Schandel and Jenness, 1994); they lack the SINNDAKSS endocytosis signal sequence (Rohrer et al., 1993).

**Fig. 2.**
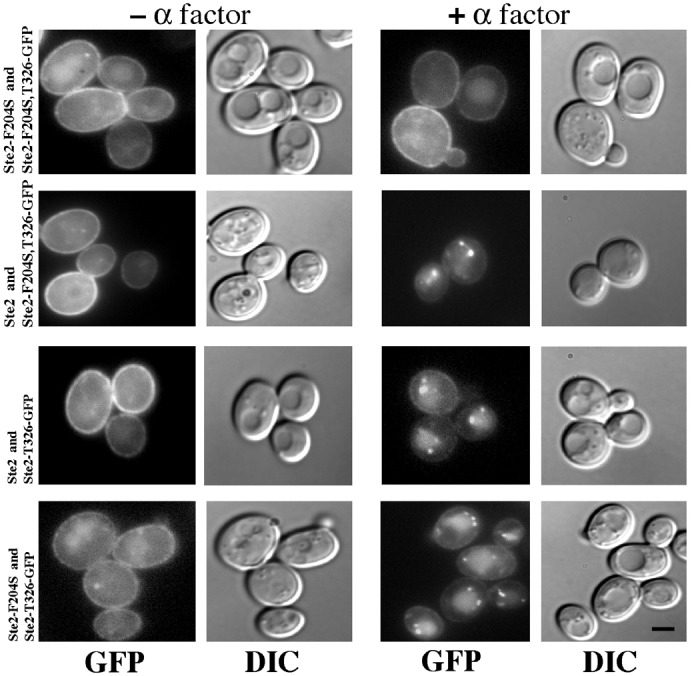
Ligand-induced internalization of GFP-tagged endocytosis-defective receptors depends on presence of full-length binding-defective receptors Ste2-F204S or full-length wild-type receptors Ste2. Cultures were treated with cycloheximide and α-factor for 15 minutes at 30°C. GFP fluorescence images and Nomarski images (DIC) are indicated below each column; first two columns are cultures lacking α-factor; the last two columns are cultures treated with α-factor. First row, cells expressed both untagged Ste2-F204S and Ste2-F204S,T326-GFP (strain DJ485-1 containing plasmid pDJ651). Second row, cells expressed both untagged Ste2 and Ste2-F204S,T326-GFP (strain DJ484-1 containing plasmid pDJ651). Third row, cells expressed both untagged Ste2 and Ste2-T326-GFP (strain DJ484-1 containing plasmid pDJ469). Bottom row, cells expressed both untagged Ste2-F204S and Ste2-T326-GFP (strain DJ485-1 containing plasmid pDJ469). Scale bar: 2 µm.

Our laboratory previously has shown that when wild-type α-factor receptors undergo ligand-mediated endocytosis, they cause a concomitant internalization of co-expressed endocytosis-defective receptors (Yesilaltay and Jenness, 2000). The defective receptors either lacked the α-factor binding site (Ste2-S184R) or lacked the endocytosis-signal sequences located in the C-terminal cytoplasmic domain of the receptor (Ste2-T326). The results were taken as evidence for endocytosis of homo-oligomeric receptor complexes. In the present study, we have extended this approach to investigate whether receptor-mediated endocytosis requires that the α-factor binding site and the endocytosis signal reside in the same receptor protein or whether the two elements can promote endocytosis even when they reside in different receptors of the oligomeric complex. We used the Ste2-F204S mutant instead of the Ste2-S184R to block α-factor binding (Fig. 1) because Ste2-F204S leads to a more pronounced defect in the α-factor response (Dosil et al., 2000). Ste2-T326 mutant receptors (Konopka et al., 1988) are truncated at residue 326 in the cytoplasmic C-terminal domain, and they lack the SINNDAKSS endocytosis signal sequence (Hicke et al., 1998; Rohrer et al., 1993). Green fluorescent protein (GFP) was fused to the C-terminus of the truncated Ste2-T326 receptors and to the truncated binding-defective Ste2-F204S,T326 receptors (Ste2-T326-GFP and Ste2-F204S,T326-GFP, respectively) in order to monitor their position by fluorescence microscopy when they are co-expressed with full-length untagged receptors (wild-type Ste2 or binding-defective Ste2-F204S). Our previous results (Yesilaltay and Jenness, 2000) showed that Ste2-T326-GFP is not subject to α-factor-induced internalization in absence of co-expressed receptors. In the present study, cells co-expressing two binding-defective receptors (Ste2-F204S,T326-GFP and Ste2-F204S) served as a negative control (Fig. 2, top row). In the absence and presence of α-factor, cells exhibited similar fluorescence both at the plasma membrane and in the vacuole. Fluorescence in the vacuole reflects the slow basal rate of constitutive endocytosis; free GFP remains intact in the vacuole after the Ste2-GFP fusion proteins have been endocytosed and the Ste2 portion of the fusion proteins has been degraded (Li et al., 1999). Yeast cells co-expressing Ste2-T326-GFP and wild-type Ste2 served as a positive control (Fig. 2, second row). Yeast cells co-expressing tagged truncated Ste2-F204S,T326-GFP and wild-type Ste2 served as a second positive control (Fig. 2, third row). In the absence of α-factor, both positive control strains showed very similar fluorescence localization. In the presence of α-factor, unlike the negative control strain, both strains showed that the fluorescence on the plasma membrane had been reduced significantly and that the fluorescence mostly appeared in internal punctate structures.

Interestingly, cells co-expressing both truncated Ste2-T326-GFP and the full-length binding-defective mutant Ste2-F204S (Fig. 2, bottom row) showed a pattern that was indistinguishable from the positive controls, indicating ligand-mediated endocytosis occurs when the α-factor binding site and the endocytosis signal reside in separate receptors. The GFP was significantly decreased on the plasma membrane and mostly appeared in the internal punctate structures when α-factor was present. We analyzed the data quantitatively in Table 1. For negative control cells expressing both Ste2-F204S,T326-GFP and full-length Ste2-F204S, α-factor did not induce the appearance of fluorescence foci, consistent with the inability of either receptor to bind α-factor. In contrast, over 80% of cells co-expressing both Ste2-T326-GFP and Ste2-F204S showed one or more foci after α-factor treatment. Similar results were observed for both of the positive controls (Ste2 expressed as Ste2-F204S,T326-GFP and Ste2-F204S expressed as Ste2-F204S,T326-GFP).

**Table 1.**
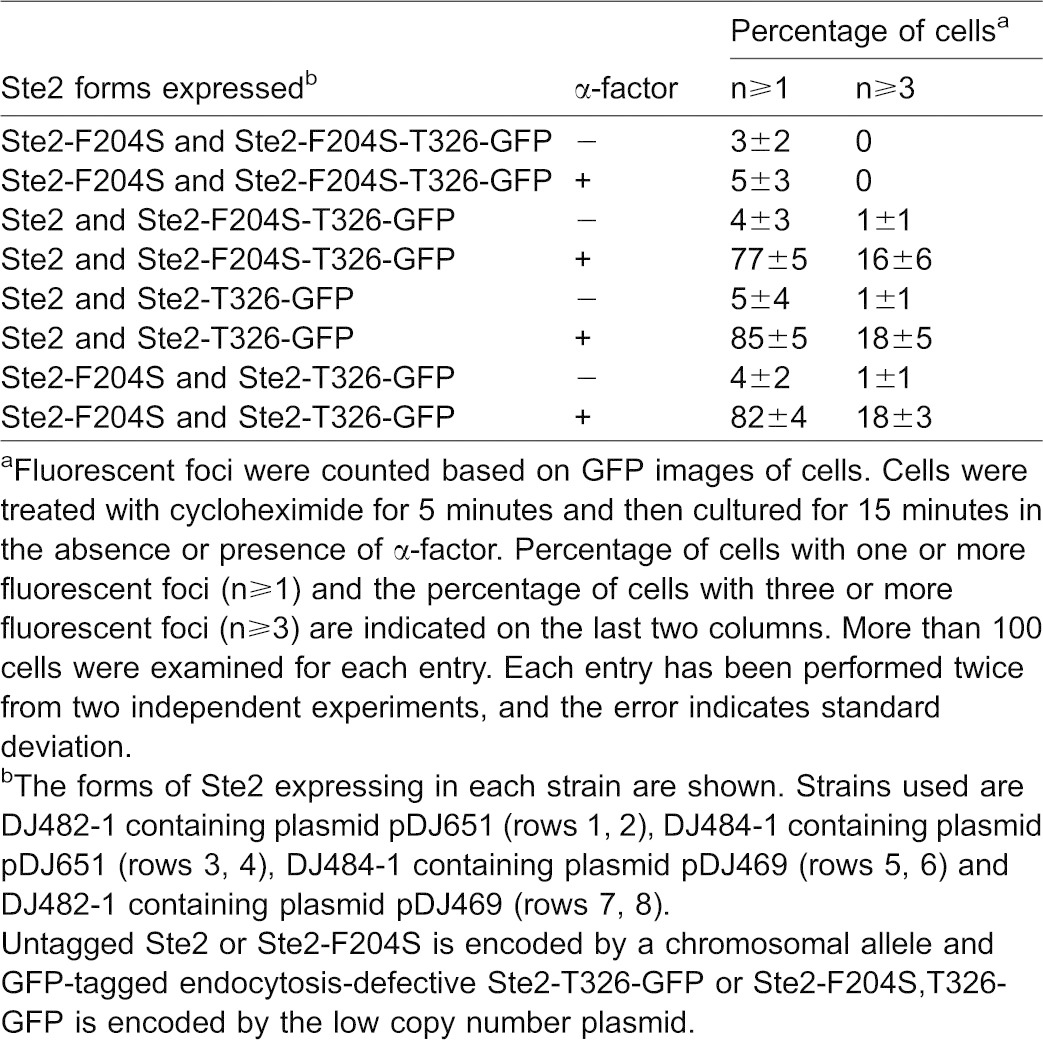
Quantitative summary of fluorescent foci induced by α-factor in the endocytosis complementation assay

These endocytosis complementation results indicated that two mutant subunits in a receptor complex can cooperate during endocytosis if one subunit provides the α-factor binding site (Ste2-T326-GFP) and another provides the endocytosis signal sequence (Ste2-F204S). We considered two models consistent with these results. In one model, the ligand-induced change in one subunit is communicated to the neighboring subunit, activating its endocytosis signal sequence. In the other model, the endocytosis machinery recognizes the ligand-induced conformational change and the endocytosis signal sequence independently. In the first model, the endocytosis signal plays an active role in ligand-induced endocytosis, whereas in the second model the endocytosis signal plays a passive role. In order to distinguish between these two models, we tested whether α-factor binding to one receptor subunit influences the conformational state of other subunits in the receptor complex.

### Conformational changes among co-expressed wild-type and mutant α-factor receptors

We employed three methods to detect α-factor induced conformational changes in binding-defective receptors when they are co-expressed with binding-competent receptors. The α-factor-induced conformational changes can be detected *in vitro* by subjecting purified plasma membranes to limited trypsin digestion in the presence and absence of α-factor (Büküşoğlu and Jenness, 1996). The influence of α-factor on the rate of specific cleavages can be monitored by western blotting. The third intracellular loop has been found to be more accessible to trypsin cleavage in the ligand occupied receptors, whereas a site near the SINNDAKSS endocytosis signal in the C-terminal domain of Ste2 is more accessible to trypsin cleavage in the unoccupied receptors (Büküşoğlu and Jenness, 1996). Differences in trypsin cleavage rates between occupied and unoccupied receptors reflect ligand-mediated changes in both the third intracellular loop and in the C-terminal domain. Recent evidence suggests that changes in accessibility of the third intracellular loop are likely to reflect movements in the flanking transmembrane domains (Umanah et al., 2011).

**Fig. 3.**
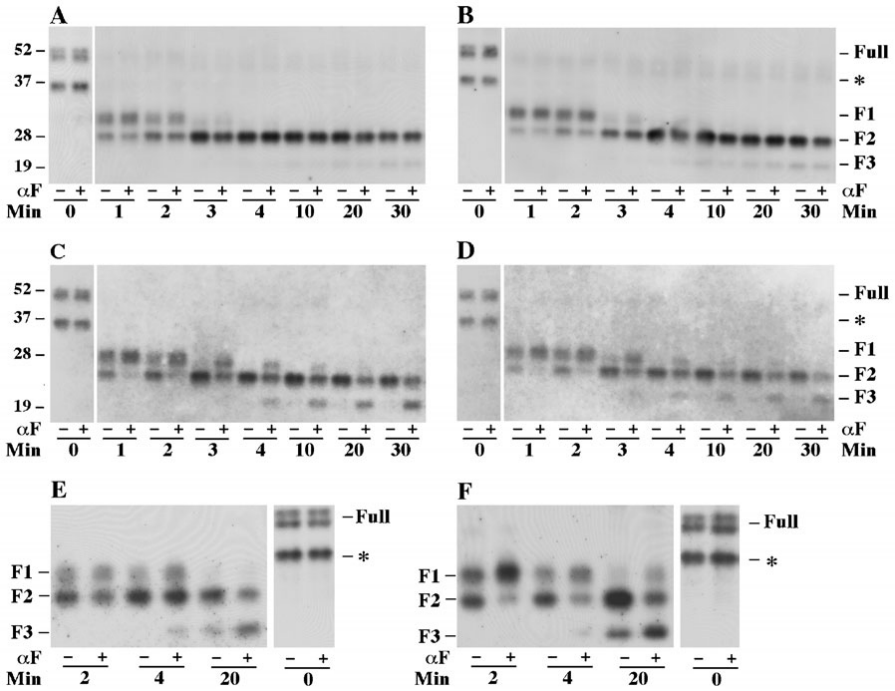
Limited trypsin digestion time course for co-expressed wild-type and T7-Ste2-F204S mutant receptors. Purified plasma membranes were incubated with trypsin in the absence (−) or presence (+) of 10^−8^ ^M^ α-factor (αF). Resulting cleavage products were analyzed by SDS-PAGE and western blotting. Digestion reaction I contained membranes from cells expressing T7-Ste2-F204S and no wild-type Ste2 (strain DJ213-7-3 containing plasmid pDJ655). Digestion reaction II contained membranes from cells expressing T7-Ste2-F204S and over-produced wild-type Ste2 (strain DJ484-1 containing plasmid pDJ655). Membranes from cells expressing HA-Ste2 (strain DJ213-6-3::pDJ281) were added to both digestion reactions as an internal control. (A) Digestion reaction I probed with anti-T7 antiserum. (B) Digestion reaction II probed with anti-T7 antiserum. (C) Digestion reaction I probed with anti-HA antiserum. (D) Digestion reaction II probed with anti-HA antiserum. (E) Control experiment showing that Ste2-F204S receptors (T7-Ste2-F204S,N25Q,N32Q) undergo qualitatively similar ligand-induced changes when the sites are partially occupied at 5×10^−6^ M α-factor (strain DJ213-7-1 containing plasmid pDJ657). (F) Binding proficient receptors (T7-Ste2-N25Q,N32Q) with 5×10^−6^ M α-factor (strain DJ213-7-1 containing plasmid pDJ656).

**Fig. 4.**
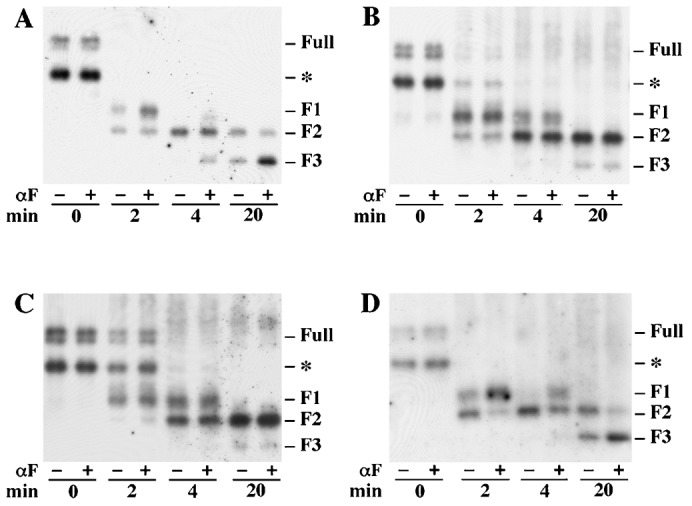
Reciprocal tests for interactions between mutant and wild-type receptors in the trypsin digestion assay. Membranes were incubated with trypsin in the absence (−) or presence (+) of 10^−8^ ^M^ α-factor (αF). Antiserum was against the T7 epitope-tag fused to the N-terminus of the receptors. Two major glycosylation sites (position 25 and 32) on T7-tagged Ste2 have been removed by genetic manipulation for experimental convenience. (A) Membranes were from cells expressing untagged wild-type Ste2 and T7-tagged Ste2-N25Q,N32Q (strain DJ213-7-3 containing plasmid pDJ656). (B) Membranes were from cells expressing untagged wild-type Ste2 and T7-tagged Ste2-F204S-N25Q,N32Q (strain DJ484-1containing plasmid pDJ657). (C) Membranes were from cells expressing T7-tagged Ste2-F204S-N25Q,N32Q (strain DJ213-7-3 containing plasmid pDJ657). (D) Membranes were from cells expressing untagged Ste2-F204S and T7-tagged Ste2-N25Q,N32Q (strain DJ482-1 containing plasmid pDJ656). The resulting cleavage products were analyzed by SDS-PAGE and immunoblotting using anti-T7 antiserum. Three major cleavage products (F1, F2 and F3) are indicated at the right. Time points for treatment of trypsin are indicated on the bottom. All untagged receptors were synthesized under the direction of the GPD transcriptional promoter.

In the present study, the trypsin accessibility assay was performed essentially as described previously (Büküşoğlu and Jenness, 1996) except that receptors contained either the HA or the T7 epitope fused at the N-terminus and purified plasma membranes were assayed instead of crude membrane preparations. Plasma membranes were digested with trypsin in the presence and absence of 10^−8^ ^M^ α-factor. At various time points, samples were withdrawn, treated with endoglycosidase H to remove N-linked carbohydrates and then resolved by SDS-PAGE. Cleavage products containing the epitope tag were detected by western blotting. At the initial time point, undigested membrane preparations contained two electrophoretic species corresponding to full-length receptor (approximately 43 kDa) and a smaller fragment (35 kDa) that apparently resulted from cleavage during membrane preparation (Fig. 3C,D). Three trypsin cleavage products were detected during the time course of the reaction. The F2 cleavage product results from cleavage in the C-terminal cytoplasmic domain at a site near the SINNDAKSS endocytosis signal, and the F1 fragment results from cleavage at a distal site in the C-terminal cytoplasmic domain. The F3 fragment results from cleavage in the third intracellular loop of the receptor. As shown in Fig. 3C,D, and consistent with our previous observations (Büküşoğlu and Jenness, 1996), the rate of cleavage at that site near the SINNDAKSS sequence was reduced when α-factor was present as judged by the slower appearance of the F2 fragment. The rate of cleavage in the third intracellular loop of the receptor was increased when α-factor was present as judged by faster accumulation of the F3 fragment. Similar results were obtained when the membranes contained receptors that were defective for both of the N-linked glycosylation sites at positions N25 and N32 in wild-type receptors (Fig. 4A). As previously reported (Mentesana and Konopka, 2001), full-length unglycosylated receptor appears as a doublet band on SDS-PAGE due to a greater level of phosphorylation. It has also been shown that mutations affecting the two sites near the receptor N-terminus of Ste2 (N25Q and N32Q) eliminated detectable N-glycosylation of receptors, and the non-glycosylated receptors retain normal function and sub-cellular location (Mentesana and Konopka, 2001).

Ste2-F204S exhibits a strong defect in α-factor binding (K_D_>10^−6^ ^M^). The mutant receptors show normal subcellular localization, and they do not alter the subcellular localization of co-expressed wild-type Ste2 receptors (Dosil et al., 2000). For the Ste2-F204S mutant to be useful, it must be capable of attaining the conformational state that is induced by α-factor. Consistent with this property, Dosil et al. found that the Ste2-F204S mutant cells were responsive to high α-factor concentration in cell division arrest (halo) assay and in α-factor induced transcription assays (Dosil et al., 2000). At high α-factor concentration (5×10^−6^ ^M^), a significant portion of the Ste2-F204S mutant receptors is expected to be ligand-occupied. We found that this ligand concentration caused trypsin to cleave the Ste2-F204S mutant receptors in manner that was qualitatively similar to its effect on wild-type receptors. After two minutes in trypsin at 5×10^−6^ ^M^ α-factor, both the Ste2-F204S mutant and the control membrane preparation showed a reduced accumulation of the F2 cleaved product relative to the F1 cleavage products; and after 20 minutes, both membrane preparations showed increased accumulation of the F3 cleavage product relative to F2 (compare Fig. 3E with Fig. 3F). These results indicate that Ste2-F204S receptors undergo ligand-induced conformational changes when the ligand-binding site is occupied at 5×10^−6^ ^M^ α-factor. At α-factor concentration of 10^−6^ ^M or less^, conformational changes in Ste2-F204S were not detected. The α-factor binding that causes a conformational change that begins to appear at 5×10^−6^ M (i.e. below the K_D_) would affect fewer than one in 500 receptors at 10^−8^ M α-factor.

When plasma membranes from cells expressing both wild-type receptors and T7-tagged Ste2-F204S mutant receptors were subjected to the limited trypsin digestion assay, binding of α-factor to the wild-type receptors was unable to influence cleavage rates of the trypsin sites in the mutant receptors. Fig. 3 compares results obtained with two different plasma membrane preparations. One preparation was from cells that expressed a normal level of the T7-tagged Ste2-F204S receptors (Fig. 3A,C), and the other preparation was from cells that expressed a normal level of T7-tagged Ste2-F204S receptors and over-expressed wild-type receptors (Fig. 3B,D). As a positive internal control, plasma membranes from cells expressing HA-tagged wild-type receptors were added to both preparations. Each of the two preparations was digested with trypsin in the presence and absence of 10^−8^ ^M^ α-factor. This concentration of α-factor induced conformational changes in wild-type Ste2 but did not induce conformational changes in Ste2-F204S. Membranes containing T7-tagged Ste2-F204S alone (Fig. 3A) and membranes containing both T7-tagged Ste2-F204S and overexpressed untagged wild-type Ste2 (Fig. 3B) resulted in trypsin cleavage patterns that were unaffected by α-factor for the 30 minute duration of the assay, even though the internal control membranes containing HA-tagged wild-type receptors resulted in trypsin cleavage patterns that were altered by the presence of α-factor (Fig. 3C,D). These results suggest that α-factor binding to wild-type Ste2 influences the conformational state; however, these ligand-induced conformational changes do not affect the conformational state of neighboring subunits in the multimeric complex that cannot bind α-factor. In other words, subunits within the same multimeric complex can apparently exist in different conformational states determined by ligand occupancy.

It has been shown that Ste2-F204S is a dominant-negative mutant that partially interferes with the functions of the wild-type Ste2 in a dose-dependent manner (Dosil et al., 2000). Recent evidence (Gehret et al., 2012) suggests that direct contact between receptors contributes to the dominance phenotype. Therefore, we considered the possibility that Ste2-F204S may interfere with ligand-induced conformational changes in wild-type Ste2 when the Ste2-F204S and Ste2 are co-expressed. Fig. 4 summarizes reciprocal tests for interactions between mutant and wild-type receptors in the trypsin digestion assay. Fig. 4 compares the trypsin cleavage assays. The mutant and wild-type receptors were defective for both N-linked glycosylation sites. Membranes from cells expressing untagged wild-type Ste2 and T7-tagged Ste2 (Fig. 4A) and from cells expressing untagged Ste2 and T7-tagged binding-defective mutant Ste2-F204S (Fig. 4B) showed tryptic digestion patterns that were consistent with the results in Fig. 3, suggesting that ligand-induced conformational changes in wild-type receptors do not influence the conformational state of co-expressed mutant receptors. Membranes from cells expressing untagged Ste2-F204S receptors and tagged receptors (Fig. 4D) resulted in ligand-induced changes in the trypsin digestion pattern that were indistinguishable from the results obtained with control membranes from cells expressing only wild-type receptors (Fig. 4A). This result indicates that Ste2-F204S does not interfere with ligand-induced conformational changes in wild-type Ste2.

**Fig. 5.**
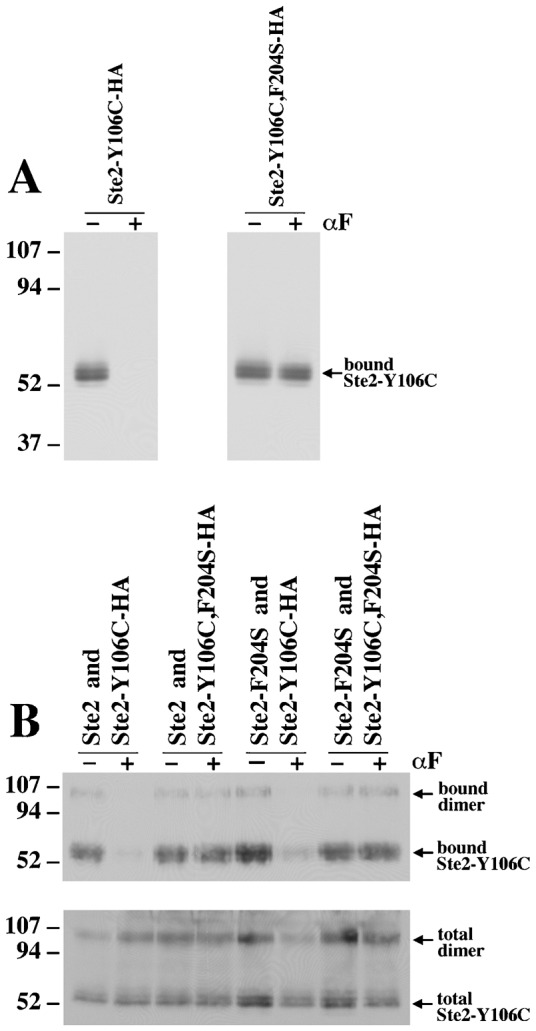
Whole-cell MTSEA-labeling of receptors containing cysteine at position 106. Cells were labeled with MTSEA in the absence (−) or presence (+) of α-factor. Detergent solubilized receptors that had been labeled with MTSEA were collected on avidin beads and analyzed by SDS-PAGE and western blotting with anti-HA antiserum. (A) Strains were DJ213-7-3 containing plasmid pDJ683 (Ste2-Y106C-HA) and strain DJ213-7-3 containing plasmid pDJ684 (Ste2-Y106C,F204S-HA). (B) Plasmids encoding Ste2-Y106C-HA or Ste2-Y106C, F204S-HA were introduced into the strains expressing wild type or Ste2-F204S under the direction of the *GPD* transcriptional promoter. Strains were DJ484-1 containing plasmid pDJ683 (Ste2/Ste2-Y106C-HA), DJ484-1 containing plasmid pDJ684 (Ste2/Ste2-Y106C, F204S-HA), DJ482-1 containing plasmid pDJ683 (Ste2-F204S/Ste2-Y106C-HA), and DJ482-1 containing plasmid pDJ684 (Ste2-F204S/Ste2-Y106C,F204S-HA). For the total protein control (lower panel), the detergent solubilized receptors were analyzed before the avidin purification step. Immunoblots are developed with anti-HA antiserum. Molecular weights of markers are indicated in kDa at the left.

Indirect methods have been used to investigate the molecular basis for the dominant negative phenotype for receptor mutants such as Ste2-F204S. Dosil et al. have found that over-production of the three G-protein subunits reverses the dominant negative phenotype even when the mutant receptors are more abundant than the wild-type receptors (Dosil et al., 1998). These results suggested that the dominant negative phenotype arises from competition between the two receptors for a limited pool of G proteins. In this view, the G proteins that recycle to receptors following α-factor activation eventually accumulate in preactivation complexes with the mutant receptor where they cannot be released by receptor activation (Dosil et al., 2000). More recently, Gehret et al. have found that the dominant negative phenotype persists even when the expression of mutant receptor is reduced to the normal level and the expression of the wild-type receptor is reduced below the normal level (Gehret et al., 2012). This finding would be inconsistent with the G-protein competition model only if the G protein pool were no longer limiting when the level of receptor expression is reduced. Interestingly, Gehret et al. also find that the dominant negative phenotype persists when the *GPA1* coding sequence is fused to the C-terminus of the wild-type receptor and provides the only source of the Gα subunit (Gehret et al., 2012). This result suggests either that the dominant negative phenotype does not result from G protein competition or that the G proteins tethered to the wild-type receptors can bind to mutant receptors within the oligomeric receptor complex. Whether or not the dominant negative phenotype of Ste2-F204S results from G-protein competition, the results of our direct assays for receptor conformation (Figs 4, 5) showed that wild-type receptors undergo ligand-induced changes in the presence of mutant receptors, suggesting that the dominant negative phenotype is a consequence of post receptor signaling activities.

### Complementation test between mutant Ste2 subunits *in vivo*

A functional test for cooperative interactions between α-factor receptors *in vivo* was explored. We reasoned that if α-factor binding to one receptor subunit can cause a neighboring subunit to assume an activated conformation then α-factor responsiveness would be restored to cells that co-express Ste2-F204S receptors and receptors that fail to couple to the heterotrimeric G protein. The *ste2-L236H* mutation affects the third intracellular loop of the α-factor receptor. The mutant receptors are partially impaired in signal transduction activity, but they retain ability to undergo α-factor induced conformational changes and ability to undergo ligand-induced endocytosis (Büküşoğlu and Jenness, 1996; Schandel and Jenness, 1994; Weiner et al., 1993). The *ste2-F204S,T326-HA* allele was used instead of *STE2-F204S* since it blocks the partial dominant negative phenotype of *STE2-F204S* at the level of receptor expression used (Dosil et al., 2000); however, at lower levels of receptor expression (Gehret et al., 2012) a truncated form of Ste2-F204S shows a dominant negative phenotype. The ability of α-factor to cause cell division arrest was judged by the α-factor halo test (not shown). When the standard halo assay was used to judge α-factor-induced cell-division arrest, the cells expressing both *ste2-F204S,T326-HA* and *ste2-L236H* alleles were found to be no more responsive than either of the single mutants alone, consistent with previous observations using full-length receptor mutants (Chinault et al., 2004). To determine whether ligand-induced changes in Ste2-L236H are propagated to Ste2-F204S,T326-HA in a shorter-term assay, we introduced a plasmid-borne, pheromone-inducible gene, *FUS1-LacZ*, into each strain and tested for β-galactosidase after α-factor exposure. Table 2 indicates that the strain expressing both *ste2-F204S,T326-HA* and *ste2-L236H* alleles was no more responsive to α-factor than the strains expressing either of the single alleles.

**Table 2.**
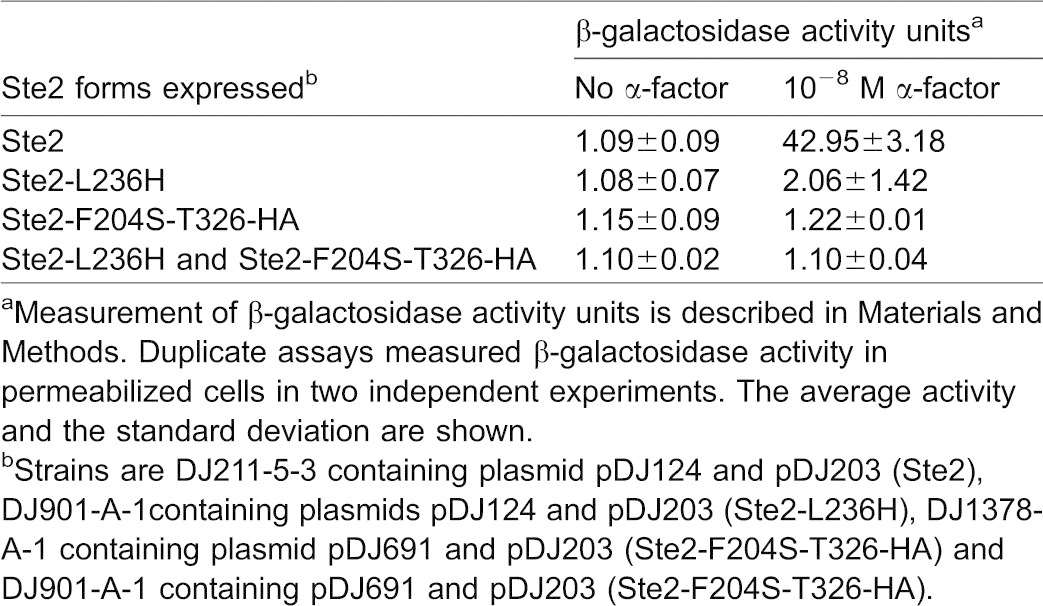
Signal complementation between different subunits of Ste2 receptors in the α-factor induced *Fus1-LacZ *assay

### Detection of conformational changes by substituted cysteine accessibility method

Whole-cell labeling with 2-[(biotinoyl) amino] ethyl methanethiosulfonate (MTSEA biotin) provides an independent assay to test whether ligand-induced conformational changes are propagated to neighboring receptor subunits. Hauser et al. first used MTSEA biotin labeling to show that the first extracellular loop of Ste2 undergoes a conformational change upon α-factor binding (Hauser et al., 2007). They showed that single cysteine residues placed at position 101 or 106 are readily accessible to solvent in the absence but not in the presence of α-factor. The decrease in solvent accessibility is not due to direct steric hindrance by the ligand since the antagonist, desTrp,Ala3-α-factor, does not significantly alter reactivity of cysteine residues at either position (Hauser et al., 2007). To determine whether α-factor binding to one subunit affects the accessibility of a cysteine residue at position 106 in an adjoining subunit, we tested whether the HA-tagged binding-defective mutant receptor, Ste2-Y106C, F204S-HA, undergoes α-factor-induced conformational changes when over-produced wild-type Ste2 is present in the same cell. In control experiments, we showed that cysteine accessibility of the binding-proficient Ste2-Y106C-HA receptor was sensitive to α-factor, whereas cysteine accessibility of the Ste2-Y106C, F204S-HA was not sensitive to α-factor when it was expressed alone (Fig. 5A). Cysteine accessibility of the Ste2-Y106C-HA receptor remained sensitive to α-factor when Ste2 or Ste2-F204S were over-expressed in the same cell (Fig. 5B). However, cysteine accessibility Ste2-Y106C, F204S-HA receptors was not influenced by α-factor when either Ste2 or Ste2-F204S were over-expressed (Fig. 5B). These results are consistent with the trypsin digestion assay (Figs 3, 4), indicating that binding-defective Ste2-F204S receptors do not undergo ligand-induced conformational changes when they are allowed to form oligomers with wild-type Ste2 receptors. In addition, failure of Ste2-F204S to impart a dominant-negative effect on ligand-induced changes in Ste2-Y106C (Fig. 5, lanes 5 and 6) is consistent with the results from the limited trypsin digestion assay (Fig. 4), further supporting the view that the partial dominant-negative phenotype of the *STE2-F204S* mutation results from G protein competition and not from direct interaction of mutant and wild-type receptors (Dosil et al., 2000).

## DISCUSSION

This study explores functional relationships among α-factor receptors when multireceptor complexes are endocytosed. We found that the complexes undergo ligand-mediated endocytosis when the α-factor binding site and the endocytosis signal sequence are located in different receptor subunits. This result implies that if ligand-mediated changes in the signal sequences are required for ligand-mediated endocytosis then the conformation associated with ligand binding must spread from the ligand-binding protomer to other protomers containing the endocytosis signal sequence. Both *in vitro* and *in vivo* assays strongly suggested that ligand-induced conformational changes in one Ste2 subunit do not affect neighboring subunits. Therefore, the recognition of the endocytosis signal sequence and the recognition of the ligand-induced conformational change are likely to be two independent events. In the train passenger metaphor, receptor protomers initiate their endocytic excursion as a group where some of the protomers can be stimulated by α-factor to help the others board the train, while only one passenger needs to carry the ticket (the endocytosis signal sequence) that is checked separately. The ability of different receptors in the complex to provide the ligand-induced conformational signal and the endocytosis signal sequence predicts that multimerization-defective mutants would be unable to cooperate in this manner. Unfortunately, we have found that α-factor receptor mutants that fail to multimerize are retained in the ER. Most of the mutant receptors that Overton and Blumer found near the cell surface (Overton et al., 2003) are probably contained in the cortical ER. We have used ER retention as phenotype to screen for additional multimerization mutants (Chang, 2009; C.-I.C. and D.D.J., unpublished data).

Endocytosis signals are contained within the cytoplasmic C-terminal domain of the receptor, since truncated receptors lacking this domain are unable to undergo both ligand-mediated and basal endocytosis, even though these truncated receptors are proficient in transducing the α-factor response signal. By generating a series of truncated receptors that removed successively larger portions of the C-terminal domain, Roher et al. identified an endocytosis signal sequence, SINNDAKSS, located near the seventh transmembrane segment (Rohrer et al., 1993). Covalent modifications of the endocytosis signal appear to be essential for its activity since endocytosis is associated with phosphorylation and ubiquitination of SINNDAKSS (Hicke et al., 1998) and since truncated receptors fused to the ubiquitin polypeptide are endocytosed constitutively even when the SINNDAKSS sequence is absent (Terrell et al., 1998). Although the signal is necessary for endocytosis, it has been unclear whether α-factor binding regulates its activity. Our previous results (Büküşoğlu and Jenness, 1996) have shown that α-factor binding affects the accessibility of trypsin to the polypeptide backbone of the receptor near the SINNDAKSS sequence, raising the possibility that ligand-induced changes in SINNDAKSS regulate its ability to participate in endocytosis.

To address the ability of ligand-induced conformational changes in one receptor to spread to neighboring protomers in the oligomeric complex, we tested whether binding-defective receptor mutants undergo conformational changes when they were co-expressed with binding-proficient receptors. Three different methods for detecting conformational change were utilized. One method was to test for allelic complementation between receptor mutants *in vivo*. Previously, no significant complementation was observed when ligand binding- and G protein coupling-defective mutant receptors were co-expressed (Chinault et al., 2004). However, since ligand-binding receptor mutants display dominant negative effects on signal transduction (Dosil et al., 1998; Leavitt et al., 1999), we repeated this strategy using truncated ligand-binding mutants which are not dominant. In agreement with the previous results, we found no allelic complementation for co-expressed ligand binding- and G protein coupling-defective mutant receptors. As a second strategy, we analyzed the changes in trypsin accessibility of receptors that occur when purified plasma membranes are exposed to α-factor (Büküşoğlu and Jenness, 1996). In plasma membranes purified from cells co-expressing wild-type and binding-defective receptors, we found that ligand-induced changes in trypsin accessibility occurred only in the wild-type receptors, again consistent with a lack of spreading of ligand-induced conformational changes. Lastly, we took advantage of the published observation (Hauser et al., 2007) that α-factor binding induces changes in solvent accessibility of cysteine residues added to the first extracellular loop of the receptor. Specifically, it was found that mutant receptors containing cysteine residues at specific positions in the first extracellular loop can be alkylated in the absence but not in the presence of ligand. We found that α-factor did not affect the alkylation of ligand-binding-defective mutant receptors containing a conformationally sensitive cysteine residue, even when the mutant receptors were co-expressed with wild-type receptors. In sum, when distant conformationally sensitive sites in the receptor were monitored (including a site near the SINNDAKSS sequence), we found no evidence for the ability of α-factor binding to one protomer to affect the conformational state of neighboring protomers within the multireceptor complex. Therefore, it is unlikely that changes in the conformation of the endocytosis signal itself are required for ligand-induced endocytosis of the receptor. However, we cannot exclude the possibility that receptors within the complex are coupled by subtle ligand-induced conformational changes, undetected by our assays.

We offer three models by which the endocytosis signal(s) may play a role in ligand-mediated endocytosis. In one model, tight packing of receptors within the multireceptor complex forms a phalanx-like structure that prevents the phosphorylating and/or ubiquitinating enzymes from gaining access to the endocytosis signals. Ligand binding to a subpopulation of the receptors may loosen the phalanx, permitting enzyme access and exposure of all endocytosis signals within the complex. In the second model, conformational changes in a subpopulation of receptors within the complex may lead to the localization of the phosphorylating and/or ubiquitinating enzymes to the complex. Concentrating these enzymes at the multireceptor complex may facilitate the modification of endocytosis signals in neighboring receptors. The regulation of protein modification by colocalizing a modifying enzyme with its protein substrates is common. For example, in the α-factor signal transduction pathway, when the Ste5 scaffolding protein binds to the activated the G_βγ_ protein, it brings the associated Ste11 MEK kinase enzyme close to the Ste20 kinase initiating the MAP kinase cascade (Pryciak, 2001). Finally, in the third model, binding of ligand may lead to the movement of the receptor complex to a subcellular compartment (e.g. a subdomain of the plasma membrane or an early endosomal compartment) where the complex is exposed to the modifying enzymes. If a subpopulation of the receptors is modified then the complex is sorted to the next endosomal compartment. If not, the complexes recycle to the initial unspecialized domain of the plasma membrane. This model is consistent with the slower ligand binding kinetics of truncated receptors lacking the endocytosis signal. Even though the equilibrium dissociation constant is unaffected k_off_ (and presumably k_on_) is markedly slower (Schandel and Jenness, 1994), consistent with the accumulation of receptor sites in an environment where diffusion of the ligand is restricted.

## MATERIALS AND METHODS

### Plasmids and yeast strains

Strains and plasmids used in this study are listed in Table 3. Single mutations in the *STE2* gene were confirmed by DNA sequencing. Integrating plasmid pDJ637 contains *TRP1* and GPD-*ste2-T58* in which GPD (glyceraldehyde-3 phosphate dehydrogenase) promoter has been fused to the first 58 codons from the *STE2* gene. It was constructed by ligating *Xho*I*/Spe*I fragment from pDJ432 with *Xho*I*/Spe*I cut pDJ429 containing the *TRP1* gene. pDJ654 that contains the wild-type *STE2* gene fused with the sequence encoding the T7 epitope was created in two steps. The first PCR product was obtained by using primer pair, ATTCCAGATATGCGTTATAACCT and ACCACCAGTCATAGAAGCCATTTTTGATTCTTGGATATGGTT, and used plasmid pDJ135 as a template. The second PCR product was obtained by using primer pair ATGGCTTCTATGACTGGTGGT and GGAATTCCCAACCATGTGGTCTGACACCAAACATAATGG, and used plasmid pDJ437 as a template. Both PCR products were gel purified and used as template in the final PCR reaction using primers ATTCCAGATATGCGTTATAACCT and GGAATTCCCAACCATGTGGTCTGACACCAAACATAATGG. The product was digested by *Mlu*I*/Hpa*I and cloned into the *Mlu*I*/Hpa*I fragment of pDJ135. Plasmid pDJ655 contains the T7 epitope-tagged version of *STE2* with a mutation at codon 204 (F204S). PCR primers CAATACACTTCCATATATGGGCAAGGATCTACCATCACTTTCGATGA and TTGCCCATATATGGAAGTGTATTGAATGGTGCTTTGACCAGGATTATA were used to construct the plasmids pDJ656 and pDJ657 that contained either T7 epitope-tagged *STE2* or *STE2*-F204S gene, and both plasmids contained mutations at amino acid position 25 and 32 (N25Q, N32Q). Two PCR products from primer pair AGAGAAAGTAGTGACAAGTGTTG and TTACTGTCTAATTGTTCTTCAGTGACTTACGCTC and primer pair CGGGACTAGTCATAAAATGTCTGATGCGGCTCCTTCAT and GTAAGTCACTGAAGAACAATTAGACAGTAAATATTTAAAATAGAG were used with template pDJ304 or pDJ451 for the second PCR products containing the Y106C or Y106C, F204S codon, respectively. This product was digested with *Hpa*I/*Cla*I and cloned into plasmid pDJ304 treated with the same enzymes to produce plasmids pDJ681 and pDJ682. Plasmids pDJ683 and pDJ684 were constructed by using a two-step PCR and cloning strategy. Plasmids pDJ681 (containing Y106C) or pDJ682 (containing Y106C, F204S) were used as the templates with primer pair ATTCCAGATATGCGTTATAACCT and CTTGGGTGGCACTAACATC to construct two PCR products (A1 and A2), and primer pair GATGTTAGTGCCACCCAAG and AGAGAAAGTAGTGACAAGTGTTG with template plasmid pDJ658 were used for another PCR product (B). The A1 and B PCR products and the A2 and B PCR products were combined and used as template together with primer pair GCATTGAGCTCGTTATCCAATGCCTGCCAA and CTTGGGTGGCACTAACATC to synthesize the PCR products containing the either full-length Y106C and Y106C, F204, respectively. These PCR products were digested with *Sac*I/*Sal*I and cloned into the plasmid pDJ481 treated with the same enzymes to produce plasmids pDJ683 and pDJ684, respectively, that carried the *LEU2* selectable marker and encoded receptors with the HA tag fused to the C-terminus. pDJ679 was constructed by cloning the *Sac*I/*Sal*I DNA fragment containing *GPD-STE2,F204S* from pDJ678 into pDJ481 treated with the same enzymes. pDJ691 was constructed by cloning the *Mlu*I/*Cla*I DNA fragment containing *STE2-F204S* from pDJ451 into pDJ469 treated with the same enzymes.

**Table 3.**
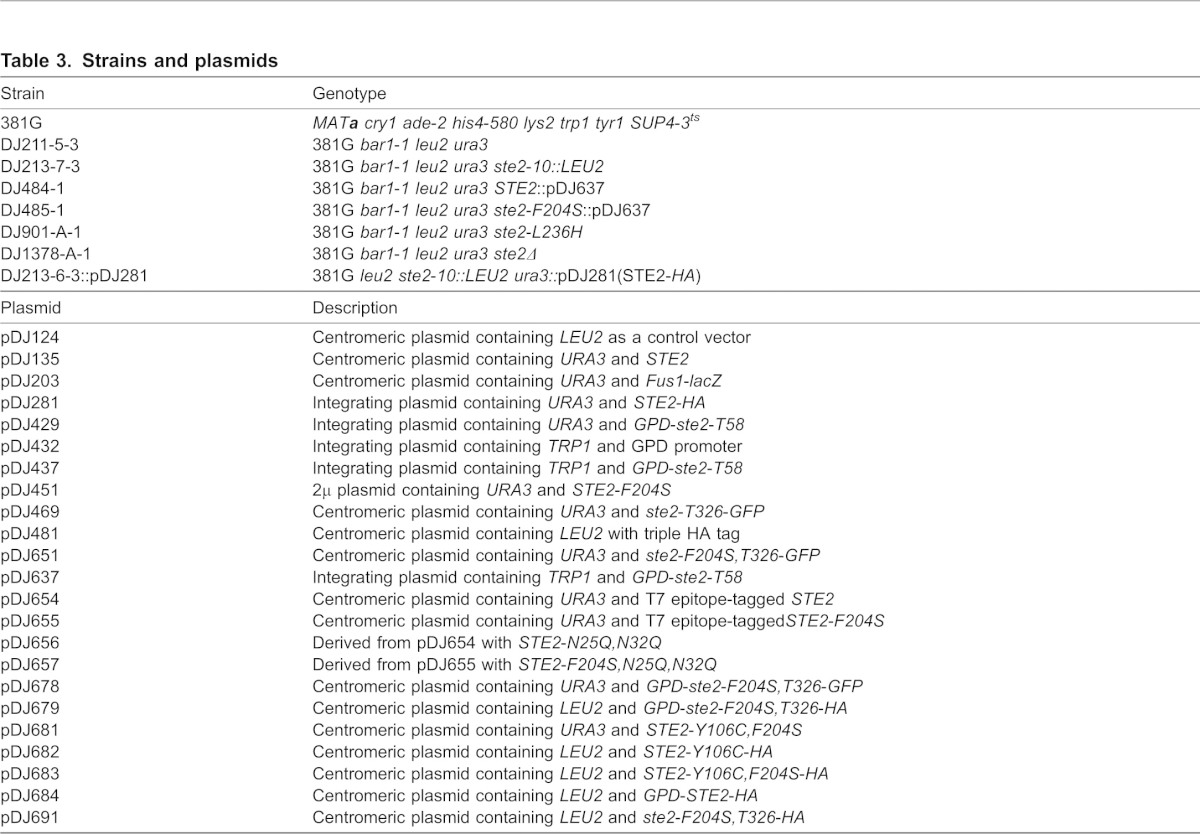
Strains and plasmids

### Fluorescence microscopy

Procedures for fluorescence microscopy were carried out as described previously (Yesilaltay and Jenness, 2000) with minor modifications. Yeast cells were grown overnight to a density of 10^7^ cells/ml in selective medium. Cells received cycloheximide (10 µg/ml) for 5 minutes before addition of α-factor (10^−7 ^M). In the parallel experiment, no α-factor was added to the culture. Cultures were incubated at 30°C for 20 minutes and terminated by the addition of the metabolic poisons, sodium azide (10 mM) and potassium fluoride (10 mM) and chilled on ice. Cells were then collected by centrifugation and suspended in ice-cold water. Epifluorescent images were obtained with a Nikon microscope equipped with a Plan Apo 100×/1.40 oil objective, a FITC-HYQ filter set and a cooled CCD camera (model ST-8I, SBIG Astronomical Instruments, Santa Barbara, CA).

### Culture media

Liquid and solid media were prepared as previously described (Hasson et al., 1994; Jenness et al., 1997).

### Antisera and reagents

Rabbit polyclonal antisera were specific for the carboxy-terminal portion of Ste2 (Konopka et al., 1988). Mouse monoclonal antibody (Anti-HA-3F10) conjugated to horseradish peroxidase (HRP) that recognizes the *influenza* hemagglutinin epitope was from Roche, Mannheim, Germany. Mouse monoclonal antibodies against T7 and conjugated to horseradish peroxidase (HRP) were from Novagen, Madison, WI. Mixtures of mouse monoclonal antibodies (Clones 7.1 and 13.1) that recognize green fluorescent protein (GFP) were from Roche, Mannheim, Germany. Peroxidase-conjugated goat anti-mouse secondary antibodies were from Sigma Chemical Co., St Louis, MO. Antibodies were used at the concentrations recommended by the supplier. Bovine serum albumin (BSA), chemiluminescence kit Super Signal and UltraLink Immobilixed Streptavidin Plus beads were from Pierce Chemical Co., Rockford, IL. MTSEA-biotin (2-[(biotinoyl) amino] ethyl methanethiosulfonate) was purchased from Biotium Co., Hayward, CA).

### Purification of plasma membranes

Yeast cells were grown in selective medium. Membranes were then resolved in renografin density gradients as previously described (Schandel and Jenness, 1994), as modified below. Renografin-60 was used to replace Renografin-76. Gradients were prepared by successively layering 2.05 ml of 34, 30, 26, 22% renografin solutions that had been prepared by diluting Renografin-60. Gradients were centrifuged for 20 h in an SW41 rotor at 30,000 rpm at 4°C. Fractions containing Pma1p were identified by resolving the proteins by SDS PAGE and staining gels with Coomassie Blue. Membranes from pooled fractions were collected by centrifugation for 90 minutes in the Ti 60 rotor at 50,000 rpm at 4°C. Membrane pellets were suspended in 300 µl buffer (50 mM TrisHCl, pH 7.5, 1 mM EDTA) and dispersed with a Dounce homogenizer. Bicinchoninic acid assay (BCA) was performed to determine the protein concentration.

### Limited trypsin digestion assay

The assay was a modification of the method of Bukusoglu and Jenness (Büküşoğlu and Jenness, 1996). Tosylsulfonyl phenylalanyl chloromethyl ketone (TPCK) treated trypsin was from Sigma. Reaction buffer for trypsin digestion assay contained 1 mM magnesium acetate, 0.1 mM dithiothreitol, 0.1 mM EDTA, 7.6% glycerol and 10 mM morpholinepropanesulfonic acid (MOPS, pH 7.0). The different time points were from separate tubes containing aliquots (2 µg) of membrane protein. Duplicate tubes contained α-factor (final concentration 10^−8^ M to 5×10^−6^ M depending on the experiment). The reaction was started by adding trypsin (final concentration 2 µg/ml). The reaction was performed at 30°C and terminated by adding 15 µl 1 N HCl. Membranes were collected by centrifugation with the Beckman Airfuge at 28 psi for 20 minutes. Membranes were then suspended at 10 µl denaturation buffer (0.5% sodium dodecyl sulfate (SDS) and 1% β-mercaptoethanol) at 42°C for 20 minutes. 10 µl endoglycosidase H mix was added to the samples and incubated at 37°C for 45 minutes. Proteins were denatured with SDS sample buffer at 37°C for 10 minutes and resolved on 12% SDS-PAGE and electrophoretically transferred to a polyvinylidene difluoride (PVDF) membrane (Millipore Corporation, Bedford, MA). The membrane was probed with horseradish peroxidase (HRP) conjugated monoclonal antibody against the T7 or the HA epitope and visualized by using the chemiluminescence kit (Pierce Chemical Co., Rockford, IL). Trypsin digestion assays using plasma membranes from cells expressing the glycosylation-defective mutant receptors were performed in a same way except that the endoglycosidase H digestion steps were omitted. At the end of the reaction, membranes were collected by centrifugation, suspended directly in the SDS sample and loaded onto SDS-PAGE.

### *Fus1-LacZ* β-galactosidase assay

Yeast cells were grown to exponential phase overnight in selective medium. Density of the culture was estimated spectrophotometrically. Cultures were diluted to the 10^7^ cells/ml, and 1 ml of cell suspension was treated with and without α-factor 10^−8^ M for 2 hours at 30°C. Each reaction was performed in duplicate. Inductions were terminated by addition of 10 µl 10 mg/ml cycloheximide. Cells were collected by centrifugation, and pelleted cells were suspended in 4 ml Z buffer (10 mM potassium chloride, 1 mM magnesium sulfate, 0.1 M sodium phosphate, pH 7). 1.5 ml cell suspension was added to 750 µl ZSB buffer (Z buffer with 50 mM β-mercaptoethanol and 0.01% SDS). Cells were permeabilized by adding 60 µl chloroform and vortexing vigorously. 200 µl of 4 mg/ml colorimetric substrate, *o*-nitrophenol-β-galactopyranoside, was added to the permeabilized cells and incubated at 28°C for 30 minutes. Reactions were terminated by adding 500 µl 1 M sodium carbonate. Cell debris was removed by centrifugation at 2,000 rpm for 10 minutes. The supernatant was transferred to a new tube to measure A_420_. Units of activity were calculated by using the formula of (1000×A_420_ of reaction)÷(A_600_ of culture×volume [in ml] of culture used×time of reaction [in min]). Units of activity were (A_420_×1000)÷(A_600_×1.5 ml×30 min) = A_420_×22.2÷A_600_.

### MTSEA-labeling, immobilization, membrane preparation and immunoblots

Whole-cell MTSEA-labeling in this study was performed essentially as described (Hauser et al., 2007) and is summarized in brief. Plasmids encoding constructs *STE2-Y106C-HA* and *STE2-Y106C,F204S-HA* were transformed into yeast strains DJ213-7-3, DJ484-1 and DJ485-1. The chromosomal *STE2* locus in yeast strains DJ484-1 and DJ485-1 was under the control of the constitutive *GPD* promoter. Cells were cultured in selective media overnight at 30°C (strain DJ213-7-3) or 34°C (strains DJ484-1 and DJ485-1). Cells were harvested at mid-log phase and processed for MTSEA biotin labeling in the presence and absence of α-factor (Hauser et al., 2007). MTSEA-biotin treated cells were lysed by vortexing with glass beads. A low speed supernatant fraction (700 × g, 5 min) was prepared, and the membranes were pelleted (15,000 × g, 30 min). Protein concentration was determined using the Bio-Rad Protein assay (Bio-Rad Laboratories, Hercules, CA). Membranes were solubilized in RIPA buffer (0.1% SDS, 1% Triton X-100, 0.5% deoxycholic acid, 1 mM EDTA in 1× PBS, pH 7.4), cleared with a high-speed spin (15,000 × g, 15 min), and adsorbed to UltraLink Immobilixed Streptavidin Plus beads (Pierce, Rockford, IL). The beads were washed extensively, extracted with SDS sample buffer (10% glycerol, 1% SDS, 0.03% bromophenol blue, 62.5 mM TrisHCl, pH 6.8, 5% 2-mercaptoethanol) at 55°C, resolved by 10% acrylamide SDS-PAGE and transferred to Immobilon™ membrane (Millipore Corporation, Bedford, MA). The blot was then probed with anti-HA antibodies. The receptors were detected by developing the blot with the West Pico chemiluminescent detection system (Pierce Biotechnology, Inc., Rockford, IL).
